# Pulmonary Tumour Embolism Complicating a Case of Leiomyosarcoma

**DOI:** 10.1080/13577149877984

**Published:** 1998-12

**Authors:** Stephen Gentle, Cyril Fisher, Neil Soni, Sue Hill, J. Meirion Thomas

**Affiliations:** 1 Chelsea and Westminster Hospital London UK; 2 Royal Marsden Hospital London UK; 3 Consultant Surgeon Chelsea & Westminster Hospital 369 Fulham Road London SW10 9NH UK

## Abstract

*Patient.* A case of peripheral leiomyosarcoma presenting with features of pulmonary thromboembolism is described.

*Discussion.* Persistence of the embolus despite triple-armed thrombolytic therapy and the presence of intravascular tumour invasion suggest the rare entity of pulmonary tumour embolism from a leiomyosarcoma.


*Sarcoma* (1998) **2**, 201–203

CASE REPORT

### Pulmonary tumour embolism complicating a case of leiomyosarcoma

STEPHEN GENTLE,[1] CYRIL FISHER,[2] NEIL SONI,[1] SUE HILL[1] & J. MEIRION THOMAS[1]

[1]*Chelsea and Westminster Hospital &* [2]*Royal Marsden Hospital, London, UK*

**Abstract**
*Patient.* A case of peripheral leiomyosarcoma presenting with features of pulmonary thromboembolism is described. *Discussion.* Persistence of the embolus despite triple-armed thrombolytic therapy and the presence of intravascular tumour invasion suggest the rare entity of pulmonary tumour embolism from a leiomyosarcoma.

**Key words**: *leiomyosarcoma, sarcoma, pulmonary embolism.*

### Case history

A 73 year-old housewife with an 8-week history of increasing shortness of breath presented with acute dyspnoea and collapse one week following tru-cut biopsy of a hard, fixed 20 cm × 10 cm swelling of the adductor compartment of the left leg. This was a leiomyosarcoma which had been slowly enlarging over the previous 2 years.

On examination she was unwell and dyspnoeic with a respiratory rate of 33 per minute. She was in sinus rhythm 74 beats per minute, blood pressure 100/50, raised JVP of 10 cm. She had a right sternal heave and a loud $P_2$ on auscultation. There was hepatomegaly.

Chest radiograph was normal and oxygen saturation on air 82%. ECG showed right bundle branch block, right axis deviation, inverted T waves in leads $V_1$ to $V_3$, Q wave and inverted T wave in lead III and S wave in lead 1.

An IVC filter was inserted and pulmonary angiography performed. This demonstrated bilateral emboli with a large embolus occluding the left inferior pulmonary artery (see Fig. 1).

A 5F Berenstein catheter was placed at the origin of this artery via a femoral approach and 5 mg of recombinant tissue plasminogen activator (rtPA) was infused as a bolus followed by 1 mg/hour over the next 24 hours. Angiograms at 6 hours and 24 hours (see Fig. 2) showed no change and therefore the rtPA was stopped, although systemic heparinisation was continued for the next 4 weeks.

V/Q scan confirmed bilateral gross perfusion defects with sparing of the left apical region and normal ventilation profile. Consecutive echo-cardiograms demonstrated a dilated right ventricle with reversed septal motion and dilated right atrium. The pulmonary artery pressure was 74 mmHg. This failed to improve, as did her oxygen saturation on

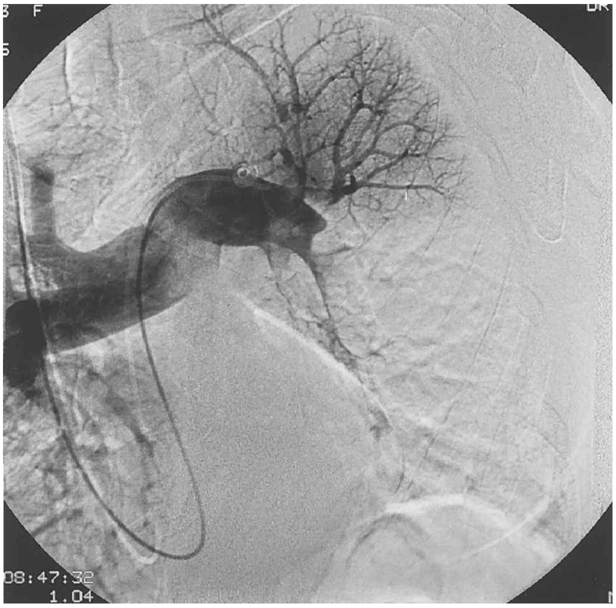

**Fig. 1.** *Angiogram to show the perfusion defect in the left lung prior to thrombolytic therapy principally affecting the left inferior pulmonary artery.*

Correspondence to: J. M. Thomas, Consultant Surgeon, Chelsea & Westminster Hospital, 369 Fulham Road, London SW10 9NH, UK.

1357-714X/98/030201-03 $9.00 © 1998 Carfax Publishing Ltd

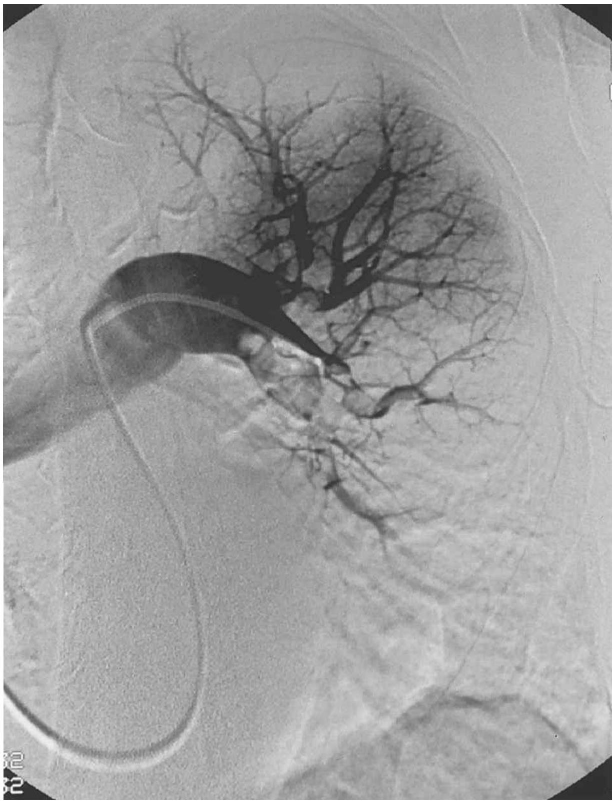

**Fig. 2.** *Angiogram to show minimal improvement in left lung perfusion following 24 hours thrombolytic therapy.*

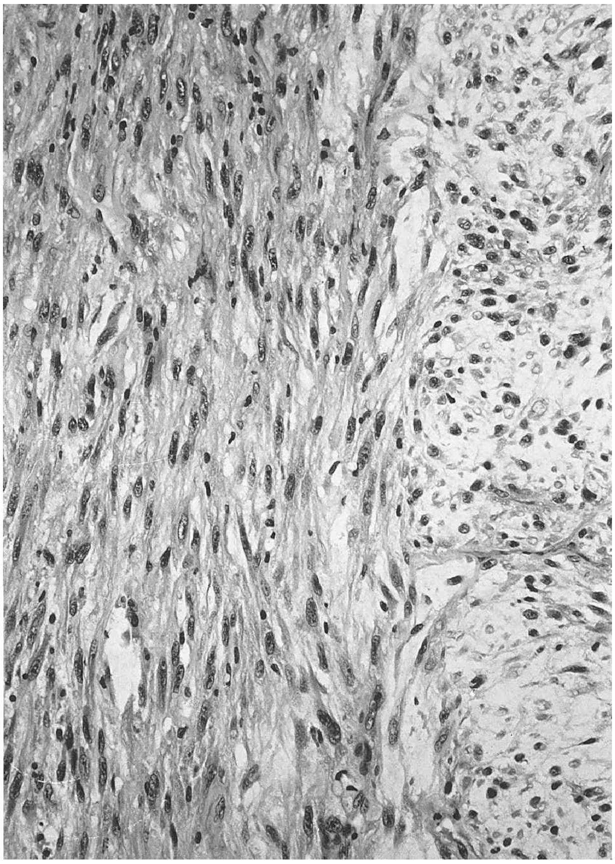

**Fig. 3.** *Leiomyosarcoma showing fascicles at right angles of spindle cells with blind-ended nuclei and paranuclear vacuoles.*

air varying from 82% to 86%. Doppler ultrasound scan of the deep venous system of the leg was negative.

Four weeks after presentation a wide excision of the tumour was performed under general anaesthetic. At operation the surrounding superficial and deep veins were filled with tumour extending proximally into the femoral veins. As much as possible of this tumour was retrieved using a Fogarty catheter. The content of the veins was proven by histology to be leiomyosarcoma and no significant thrombus was found.

Histological examination demonstrated well-circumscribed nodules of focally myxoid spindle cell sarcoma composed of fusiform cells with blunt-ended hyperchromatic nucleii and eosinophilic cytoplasm. The appearance was consistent with a diagnosis of leiomyosarcoma of intermediate grade. Many of the tumour lobules were growing within the vessels.

Excision was complete but unfortunately the patient died 5 weeks later without improvement in her cardiovascular status and post-mortem examination was refused.

### Discussion

The patient presented with clinical and radiological features of recurrent massive pulmonary thromboembolism. The evidence is indirect but we suggest that the pathophysiology in this case was pulmonary tumour embolism from the peripheral leiomyosarcoma.

The embolus was totally refractory to the triple-armed therapy comprising an IVC filter; infusion of 29 mg rtPA/24 hours directly on to the site of the main perfusion defect; 4 weeks systemic heparinisation. The efficiency of this regimen in the management of pulmonary embolism has been verified by Goldhaber *et al.*[1] who reported clot lysis in 34 of 36 patients treated with rtPA only using up to 90 mg over 6 hours infused peripherally. A lower dose was used in the presented case but was selectively administered to the site of obstruction. Rosenthal *et al.*[2] use a similar triple-armed protocol and found rtPA showed a more rapid improvement compared with streptokinase.

Winterbauer *et al.*[3] conducted a retrospective analysis using clinical records and post-mortem findings of 366 cases of renal cell carcinoma, hepatocellular carcinoma, choriocarcinoma, gastric and breast carcinoma and found an incidence of pulmonary tumour embolism of 26% with clinically significant emboli of 8.3%. Renal cell tumour, hepatocellular carcinoma and choriocarcinoma characteristically invade major vessels. Hepatic metastases appeared important in the incidence of pulmonary tumour embolism of gastric and breast carcinoma doubling the incidence in the former (23% compared with 10%) and trebling the incidence in the latter (27.5% compared with 11%). The authors (3) argue that the frequency of invasion of the central and small hepatic veins in these metastases accounts for this difference. This illustrates two points: that pulmonary tumour embolism occurs more frequently than is clinically recognised and is more common in tumours that invade veins. The findings of macroscopic venous invasion at operation and histological confirmation of intravascular leiomyosarcoma emboli therefore supports pulmonary tumour embolism as the pathophysiology in this case. An alternative explanation for pulmonary tumour embolism in our patient is that the leiomyosarcoma arose from the vein wall and extended into the surrounding soft tissue rather than the other way round.

Leiomyosarcomas are generally locally invasive and tend to compress rather than invade major vessels, unless they arise from the vessel walls. Relatively few of the low-grade tumours metastasize; about 50% of the high-grade tumours will do so. In this case we assume metastasis is by microembolisation rather than by extensive tumour permeation. A literature search identified four cases which presented with features of pulmonary embolus but were subsequently identified as leiomyosarcomas arising from the pulmonary arteries themselves (Schlecht *et al.*,[4] Reinbold *et al.*,[5] Madu *et al.*[6] and Promisloff *et al.*[7] Arbeit *et al.*[8] described a case in which an extensive retroperitoneal leiomyosarcoma with anaplastic elements invading the inferior vena cava was presumed to have embolised during surgical excision. There were no clinical features of pulmonary embolisation post-operatively and investigations were normal until 16 months later when, despite prophylactic chemotherapy and radiotherapy, a right hilar mass, identified as leiomyosarcoma, developed. The patient died 4 months later after pneumonectomy with widespread metastases. Demoulin *et al.*[9] described a case of leiomyosarcoma arising from the inferior vena cava and extending from 10 cm below the renal vessels along the entire length of the inferior vena cava with extension into the right atrium, ventricle and pulmonary infundibulum which presented with features of pulmonary embolism. Although tumour fragments were found in the peripheral branches of the pulmonary arteries at post-mortem the patient had previously undergone a Trendelenburg operation and required intraoperative defibrillation which may have fragmented the tumour. The preoperative features of pulmonary embolism may have been caused by tumour extension rather than true embolisation.

What is unusual, though not unique, about this case is the extensive permeation of tumour throughout the venous system which resulted in macroscopic tumour embolisation into the pulmonary arterial tree.

In cases of established neoplasia it may be worth considering intraluminal biopsy at the time of angiography if a large pulmonary embolus is refractory to direct thrombolysis.

### References

1 Goldhaber SZ, Baughan DE, *et al.* Acute pulmonary embolism treated with tissue plasminogen activator. *Lancet* 1986; 2:886–9.
2 Rosenthal D, Evans RD, Borrero E, Larris PA, Clark MD, Daniel WW. Massive pulmonary embolism: triple-armed therapy. *J Vasc Surg* 1989; 9(2):261–70.
3 Winterbauer RH, Elferbin IB, Ball Jr WC. Incidence and clinical significance of tumour embolization to the lungs. *Am J Medicine* 1968; 45:271–90.
4 Schlecht L, Bittner RC, Schnoy N, Loddenkemper R, Felix R. Leiomyosarcoma of the pulmonary artery. *Aktuella Radiol* 1996; 6(2):105–7.
5 Reinbold WD, Moller-Hartmann W, Korffer R, Raute-Kreinsen U. Emboliform sarcoma of the right pulmonary artery. *Radiloge* 1995; 35(7):476–80.
6 Madu EC, Taylor DC, Durzinsky DS, Fraker Jr TD. Primary intimal sarcoma of the pulmonary trunk simulating pulmonary embolism. *Am Heart J* 1993; 125(6):1790–2.
7 Promisloff RA, Segal SL, Lenchner GS, Cichelli AV, Wendell G, Aaronson G. Sarcoma of the pulmonary artery. *Chest* 1988; 93(1):207–8.
8 Arbeit JM, Flye MW, Mundinger Jr GH, Webber BL. Latent pulmonary embolus from a retroperitoneal sarcoma. *Cancer* 1980; 46(6):1492–8.
9 Demoulin JC, Sambon Y, Baudinet V, Beajean M, Jenkens JM, Delvigne J. Leiomyosarcoma of the inferior vena cava; an unusual cause of pulmonary embolism. *Chest* 1974; 66(5):597–9.
